# Xylanase Superproducer: Genome Sequence of a Compost-Loving Thermophilic Fungus, *Thermomyces lanuginosus* Strain SSBP

**DOI:** 10.1128/genomeA.00388-13

**Published:** 2013-06-20

**Authors:** Nokuthula Peace Mchunu, Kugen Permaul, Ahmad Yamin Abdul Rahman, Jennifer A. Saito, Suren Singh, Maqsudul Alam

**Affiliations:** Durban University of Technology, Department of Biotechnology and Food Technology, Durban, South Africaa; Advanced Studies in Genomics, Proteomics and Bioinformatics, University of Hawaii, Honolulu, Hawaii, USAb; Centre for Chemical Biology, Universiti Sains Malaysia, Bayan Lepas, Penang, Malaysiac

## Abstract

We report here the draft genome sequence of *Thermomyces lanuginosus* strain SSBP, which was isolated from soil in South Africa. This fungus produces the largest amount of xylanase ever reported in the literature.

## GENOME ANNOUNCEMENT

*Thermomyces lanuginosus* is a thermophilic fungus that has the ability to degrade plant biomass and produces the largest amounts of hydrolyzing enzymes (1). Because of this ability, this fungus has been identified as one of the organisms that can have various industrial applications. Thus, sequencing of the *T. lanuginosus* genome is important because this will provide necessary information needed for better industrial usability of this fungus.

Whole-genome sequencing was performed using Roche 454 and Illumina paired-end sequencing strategies. The Roche 454 sequencing strategy with single ends and paired ends was used to construct a genomic library, and a Solexa sequencing strategy was used to construct a paired-end and mate-paired genomic library. We are reporting a 23.3-Mb *T. lanuginosus* genome sequence that was created by *de novo* assembling of 98% of the sequencing data generated using next-generation sequencing technology. The repeat reads were <0.5% of the total reads generated. The assembly has long-range continuity, with an *N*_50_ scaffold size of >4 Mb. Most of the assembly (90%) is contained in the 6 largest scaffolds and the rest in 24 smaller scaffolds with lengths of <10 kb.

The proteome that was predicted from this assembly had a total of 5,105 genes, which is less than that of other filamentous fungi, with 83 tRNA genes (2–6). The genome was annotated using a modified version of Maker (7). The GC content of the whole genome was calculated to be 52.14%, while coding regions had a significantly higher GC content, at 55.6%. As this thermophilic fungus is a wood-degrading fungus, the CAZy family protein was analyzed in depth. Carbohydrate-active enzymes (CAZy) have the ability to cleave or add monomers to polysaccharides, and because of this characteristic many of these fungi have major importance in the biotechnology industry. A total of 224 predicted proteins that were identified using the CAZy database belong to this group of proteins (8).

Mechanisms of thermal adaptation by this fungus, especially in relation to the genomic material, were suggested by the presence of several DNA-related pathways. *T. lanuginosus* has a ubiquitin degradation pathway which plays a crucial role in the responses to various stressors, such as nutrient limitation, heat shock, and heavy metal exposure (9). *T. lanuginosus* is a thermophilic organisms that generally grows on dead woody material. The ubiquitin degradation system may be essential for adaptation during rising temperatures in composting materials. *T. lanuginosus* is also capable of histone acetylation/deacetylation and poly ADP-ribosylation and contains high numbers of methylases. Histone acetylation/deacetylation and methylation play important roles in packing and condensation of DNA (10). Poly ADP-ribosylation is the addition of one or more ADP-ribose moieties to a protein (11). It plays an important role in cell signaling and the control of many cell processes, including DNA repair and apoptosis (12, 13). All of these DNA condensation machinery and repairing mechanisms make this fungus well adapted to living and thriving at high temperatures.

### Nucleotide sequence accession number.

This Whole-Genome Shotgun project has been deposited at DDBJ/EMBL/GenBank under the accession number ANHP00000000. The version described in this paper is the first version.
